# Digital patient decision aids for cancer prevention in socially disadvantaged women: A scoping review protocol

**DOI:** 10.1371/journal.pone.0359332

**Published:** 2026-10-14

**Authors:** Emmanuelle Lüthi, Océane Pittet, Joanna Montagnese, Romain Freund, Kevin Selby, Véronique S. Grazioli, Thomas Brauchli, Marie-Anne Durand

**Affiliations:** 1 Department of Ambulatory Care, Unisanté, University Center for Primary Care and Public Health & University of Lausanne, Lausanne, Switzerland; 2 Department of Epidemiology and Health Systems, Unisanté, University Center for Primary Care and Public Health & University of Lausanne, Lausanne, Switzerland; 3 Department of Vulnerabilities and Social Medicine, Unisanté, University Center for Primary Care and Public Health & University of Lausanne, Lausanne, Switzerland; 4 Research Support Unit, Unisanté, University Center for Primary Care and Public Health & University of Lausanne, Lausanne, Switzerland; 5 The Dartmouth Institute for Health Policy & Clinical Practice, Dartmouth College, LebanonUnited States of America; Independent Medical Researcher and Writer, UNITED KINGDOM OF GREAT BRITAIN AND NORTHERN IRELAND

## Abstract

This scoping review aims to map and analyze the existing evidence on digital Patient Decision Aids (PtDAs) for cancer prevention targeting socially disadvantaged women. Cancer is a leading cause of premature mortality among women. Cancer prevention strategies can effectively reduce cancer incidence and mortality. Socially disadvantaged women face lower screening rates and higher mortality. Effective cancer prevention requires informed decision-making. One approach is the use of digital PtDAs. They represent a promising innovation for this population. However, their characteristics, development process, and evaluation for cancer prevention targeting this population remain underexplored. This scoping review will include digital PtDAs designed to meet the needs of socially disadvantaged women aged 18–75 in the context of cancer prevention and meeting the minimum criteria established by the International Patient Decision Aid Standards. We will include studies and sources covering quantitative and qualitative research, and gray literature. This scoping review will follow the JBI methodology for scoping reviews. A comprehensive three-step search will be conducted across MEDLINE, CINAHL, Embase, Web of Science, Cochrane databases, Google Scholar and gray literature sources. All studies and sources available in any language will be considered. Citations will be imported into Rayyan for screening. Two independent reviewers will conduct study selection, data extraction, and analysis. Data will be summarized using tables and descriptive content analysis to identify characteristics, development processes, and evaluation methods of digital PtDAs. The review will be reported in accordance with PRISMA-ScR guidelines. Findings will be submitted for publication in an international peer-reviewed journal and presented at national and international conferences focused on cancer prevention, shared decision-making, digital health or socially disadvantaged women. We will share findings with healthcare professionals, public health and cancer prevention stakeholders, and socially disadvantaged communities and their network. This protocol has been registered with the Open Science Framework (https://osf.io/f3428/).

## Introduction

Cancer prevention strategies, such as human papillomavirus (HPV) vaccination and screening tests, can effectively reduce cancer incidence and mortality [1–3]. However, socially disadvantaged populations tend to have lower vaccination uptake [4], lower screening rates [5–7], and higher exposure to tobacco [8], obesity [9], and inactivity [10]. They also face higher mortality rates for nearly all cancer-types [11], but particularly for lung cancer [12], breast cancer [13,14], cervical cancer [15] and colorectal cancer [16]. Cancer prevention offers benefits but sometimes also carries risks, making informed decision-making essential, aligned with people’s values and preferences. Providing evidence-based information is important to prevent overuse and overdiagnosis. Shared decision-making (SDM), through the use of patient decision aids (PtDAs), is a solution to promote access to clear and accessible information about available prevention options. PtDAs are defined according to these three characteristics: 1) “They provide facts about a person’s condition, the options and their features”. 2) “They help people to clarify their values (the features that matter most to them).” 3) They help people to share their values and to discuss the option with their healthcare professionals and others, so a course of action can be planned that matches their values [17,18]. The use of PtDAs can, in turn, enable discussions with their healthcare professionals to achieve informed preferences and decisions. Although better-quality decisions do not always increase screening uptake, they remain ethically important.

Based on a rapid review of the literature that did not specifically focus on women, we identified a digital chatbot-based PtDA to improve participation in cervical cancer screening among socially disadvantaged women in France, developed by Selmouni et al. [19] and a conversational agent to promote informed decision-making regarding prostate cancer screening among African-American men, developed by Owen et al. [20]. The evaluation of Owen’s tool showed significant improvements in prostate cancer knowledge, self-efficacy in informed decision-making, and technology usage. The evaluation of Selmouni’s chatbot PtDA for cervical cancer screening suggested that the chatbot not only enhanced knowledge about cervical cancer screening but also significantly boosted motivation to engage in screening practices. Users—including those with limited digital literacy—found the Selmouni’s chatbot innovative, user-friendly, and informative [21].

However, people vary in their capacity to engage in SDM. Social disadvantage can hinder participation in both SDM and use of PtDAs, thereby contributing to disparities in cancer prevention uptake and outcomes [22,23]. Socially disadvantaged individuals face substantial barriers to accessing healthcare services [24,25]. They are more likely to have lower health literacy levels and/or lower educational attainment, requiring adapted information strategies [26]. In addition, psychological barriers such as fear, shame and a lack of cultural sensitivity among healthcare professionals can further impede access to preventive care [27].

Although PtDAs have the potential to support informed decision-making and reduce inequities, many are not designed to address the specific needs, preferences, and contextual realities of socially disadvantaged populations. As a result, their accessibility, relevance, and effectiveness for these groups may be limited.

To date, it remains unclear how digital cancer prevention PtDAs have been developed, adapted, and evaluated for socially disadvantaged women.

A preliminary search of MEDLINE, the Cochrane Database of Systematic Reviews and JBI Evidence Synthesis was conducted, and no relevant systematic reviews or scoping reviews (published or underway) were identified. Further, a rapid review of PtDAs available in French for cancer prevention was conducted in 2024. This rapid review did not specifically focus on socially disadvantaged women. Twelve digital PtDAs were identified [28]. Consequently, there is a need to map and synthesize the existing evidence on digital cancer prevention PtDAs targeting this population, including their characteristics, development process, and reported impacts. A scoping review is the most appropriate methodology for mapping the existing literature and addressing this important knowledge gap. This scoping review aims to address this important gap in the literature.

The research questions: What are the characteristics, development process and impact of digital PtDAs for cancer prevention targeting socially disadvantaged women?

This scoping review aims to map the body of evidence on digital PtDAs for cancer prevention (characteristics, design and impact) targeting socially disadvantaged women, across a range of healthcare contexts.

This review question focusses on three main objectives:

1. To identify and describe the characteristics of existing digital PtDAs for socially disadvantaged women in cancer prevention, using the International Patient Decision Aid Standards (IPDAS) minimal criteria [29] and the Template for Intervention Description and Replication (TIDieR) [30].
2. To examine the development processes, including stakeholder involvement and use of frameworks or guidelines.
3. To summarize the evaluation methods and the type of impact reported.

## Materials and methods

The proposed scoping review will be designed and conducted in accordance with the JBI methodology for scoping reviews [31] and reported according to the PRISMA extension for scoping reviews (PRISMA-ScR) guidelines (S1 File) [32]. Following the development of the search strategy (September 2025 to January 2026), study and source-of-evidence selection will be conducted between February and August 2026. Data extraction, analysis and synthesis are expected to be completed within the subsequent 12 months. This review protocol has been registered in the Open Science Framework (OSF) registry (registration number: f3428) [33].

### Eligibility criteria

This scoping review will consider a broad range of evidence sources to comprehensively map the literature on PtDAs for socially disadvantaged women around cancer prevention. Eligible sources are described in Table 1.

**Table 1:** Study eligibility criteria.

Domain	Included	Excluded
Participants	• Reports on PtDAs and PtDAs themselves explicitly designed to meet the needs of socially disadvantaged women according to the PROGRESS-Plus framework [34], which refers to the place of residence, race/ethnicity, culture, language, occupation, gender/sex, religion, education, socioeconomic status, and social capital. • Women at average risk of cancer, i.e., without hereditary syndromes or symptoms of cancer. Age: 18–75 years old to reflect the different recommended screening thresholds in Switzerland for the cancers prioritized in this review (breast, cervical, colon and lung cancer). • Adult population to avoid including parents or guardians, who would influence the dynamic of shared decision-making.	Articles reporting less than 50% of data from adult socially disadvantaged women (by mentioning sex and/or gender categories) or not conducting a separate analysis for this group [35].
Concept or intervention	• Digital refers to any PtDA that is delivered through digital technologies and is accessible remotely (access to a digitally hosted tool – via a device – such as a phone, tablet, computer or similar). • Designed for use on computers, mobile devices (smartphones, tablets), or other personal digital devices that allow access outside of a single fixed location. • If a tool is currently paper-based but is described in the literature as in the process of being digitized, it will be included if sufficient detail about the planned digital features is provided. The final decision on borderline cases (e.g., web-based information sheets that partly meet PtDA criteria) will be made through discussion and consensus among the review team. • Any type of chatbots or conversational agents (rule-based, AI-driven, and large language model (LLM)-based systems).	PtDAs that are available only in non-digital formats (e.g., printed materials, PDF files) and cannot be accessed remotely through a computer, mobile device, tablet, or other personal digital device.
Outcome	• Studies must describe the development or evaluation, of PtDAs designed to support patients in making informed choices in cancer prevention. According to the IPDAS Collaboration [29,36,37], a PtDA must, at minimum: (1) clearly state the decision to be made; (2) provide evidence-based information about a health condition, the options, associated benefits, harms; (3) help patients clarify the importance they place on each option’s potential benefits and harms (implicit or explicit).	Tools that do not fulfill the minimal IPDAS definition of a PtDA will be excluded, such as interventions that provide general education only, without linking it to a specific healthcare decision; or are designed primarily to promote treatment adherence or to obtain informed consent for a recommended option rather than to support a deliberative choice among alternatives.
Context	• All studies conducted in any healthcare setting or geographic location. • PtDAs addressing primary and secondary cancer prevention (all cancer types). • Primarily include screening processes.	• PtDAs addressing primary prevention as behavior changes that can indirectly prevent cancer (i.e., smoking cessation or weight loss). • Secondary cancer prevention as the interventions leading to the discovery and control of cancerous or precancerous processes while localized.
Study Design	Quantitative • Experimental and quasi-experimental designs including randomized controlled trials, non-randomized controlled trials, before and after studies and interrupted time-series studies. • Analytical observational studies including prospective and retrospective cohort studies, case-control studies and analytical cross-sectional studies. • Descriptive observational study designs including case series, individual case reports and descriptive cross-sectional studies for inclusion. Qualitative research • with insights into the development, implementation, or experiences related to digital PtDAs will be eligible. This includes studies using methodologies such as phenomenology, grounded theory, ethnography, qualitative description, action research, and feminist research. Gray literature and non-empirical sources, such as policy documents, websites, and organizational reports, particularly those offering valuable context or information on the design, features, or use of PtDAs in practice. Standalone PtDAs without accompanying research to comprehensively map the available tools.	• Studies for which neither the full text nor the PtDA can be obtained despite reasonable efforts will be excluded, as insufficient information would be available to assess eligibility or extract data. • Studies published from database inception will be included to ensure a comprehensive search that captures the emergence and evolution of digitalization, including the early development of web-based and mobile PtDAs.
Publication Language	All	None

To access existing AI tools, we will first contact the author by email (first, second, last, and corresponding authors). If we do not receive a response within a week, we will send a follow-up email. If no response is received within two weeks, the request will be considered closed.

### Search strategy

A three-step search strategy aimed to locate both published and unpublished studies. First, an initial limited search of MEDLINE ALL (Ovid) and CINAHL (EBSCO) was undertaken to identify articles on the topic. The words contained in the titles and abstracts of relevant articles, and the Medical Subject Headings (MeSH) and index terms used to describe the articles were used to develop a full search strategy in MEDLINEALL (Ovid) (S2 File). The search strategy has been developed by the research team with the help of an information specialist. The search strategy, including all identified MeSH terms and keywords, was adapted for each database and/or information source. The databases to be searched included MEDLINE ALL (Ovid), CINAHL Complete (EBSCO), Embase.com, Web of Science (Core collection), Cochrane Database of Systematic Reviews (Wiley) and Cochrane Central Register of Controlled Trials (Wiley). To ensure a comprehensive coverage, gray literature and online resources such as the online inventory of Decision Aids developed by the Ottawa Hospital Research Institute [38] and the MAGIC app (https://www.magicevidence.org/magicapp/) were included. The first five pages of Google search results were screened, along with relevant websites of health organizations, government agencies, and patient advocacy groups. Additionally, the first 200 results from a targeted Google Scholar search were reviewed. The search strategy for Google Scholar was developed and refined in collaboration with the information specialist.

To identify any additional relevant studies not captured in the database searches, we conducted a backward citation search of the reference lists of all included full-text articles and relevant reviews. This backward citation searching helped capture additional studies that meet inclusion criteria and relevant gray literature or earlier foundational works. Any newly identified citations were screened according to the same eligibility criteria used in previous steps.

Studies published in any language will be included. Studies published from database inception will be included to ensure a comprehensive search that captures the emergence and evolution of digitalization, including the early development of web-based and mobile PtDAs.

### Study/Source of evidence selection

Following the search, all identified citations will be collated and uploaded into Rayyan [39] and duplicates removed. Following a pilot test, titles and abstracts will be screened by two independent reviewers for assessment against the inclusion criteria for the review. Relevant sources selected and discussed by the two independent reviewers will be retrieved in full and their citation details imported into Rayyan. The full text of selected citations will be assessed in detail against the inclusion criteria by two independent reviewers. Reasons for the exclusion of sources of evidence at full text that do not meet the inclusion criteria will be recorded and reported in the scoping review. Any disagreements that arise between the reviewers at each stage of the selection process will be resolved through discussion, or with an additional reviewer/s. The results of the search and the study inclusion process will be reported in full in the final scoping review and presented in a PRISMA flow diagram [32].

For each PtDA, we will seek to obtain a copy of the tool itself, along with any associated publications or documentation describing its characteristics and features. If access to the tool is not possible, data will be extracted based solely on the associated publication or report. Standalone PtDAs without accompanying research will also be included to comprehensively map the available tools. This approach will provide a realistic mapping of the available evidence based on the information accessible to the scientific community.

### Data extraction

Data will be extracted from all sources of evidence included in the scoping review by two independent reviewers using a standardized data extraction form. The extracted data will include specific details about the participants, concept, context, study methods, and key findings relevant to the review questions. We will use the data extraction form provided in Appendix 10.1 of the JBI Manual for Evidence Synthesis – Scoping Reviews chapter [31] as well as IPDAS minimal criteria [29] and the Template for Intervention Description and Replication (TIDieR) [30] (S3 File.). These forms will be piloted on a small number of sources to ensure consistency and clarity. They may be modified and revised as needed during the data extraction process; any changes will be documented in the final review. Disagreements between reviewers will be resolved through discussion or consultation with a third reviewer. When required, authors of sources may be contacted to obtain missing or additional information.

### Data analysis and presentation

In this scoping review, the extracted data will be collated, summarized, and reported using tabular presentations and descriptive content analysis. Tables will display key information such as the frequency of study characteristics, features of digital PtDAs, implementation strategies, and reported outcomes related to usability, accessibility, acceptability, feasibility, and patient-reported experiences. We will do a descriptive content analysis to identify recurring themes, patterns, and gaps within the literature.

The review will be reported in accordance with the PRISMA-ScR guidelines [32] to ensure transparency and methodological rigor.

### Patient and public involvement

This scoping review is the first phase in a broader four-steps project aimed at developing a digital PtDA for cancer prevention for socially disadvantaged women. To ensure meaningful engagement of the target population throughout the research process, two citizen partners with lived experience of migration are members of the project steering committee. They contribute to project governance through monthly meetings, during which they co-review study materials, participate in key decisions regarding project identity and communication (e.g., project name and logo), co-develop participant recruitment strategies, and co-facilitate meetings with study participants. Their active and sustained involvement helps ensure that the project remains relevant, acceptable, and responsive to the needs and preferences of the intended users.

## Discussion

We conducted this scoping review to map and synthetize the body of evidence on digital PtDAs for cancer prevention (characteristics, design and impact) targeting socially disadvantaged women. Our review question focused on three main objectives: a) To identify and describe the characteristics of existing digital PtDAs for socially disadvantaged women in cancer prevention, using the IPDAS minimal criteria [28] and the TIDieR checklist [29]; b) To examine the development process, including stakeholder involvement and use of frameworks or guidelines; c) To summarize the evaluation methods and the type of impact reported for these PtDAs.

Currently, women, especially those from socially disadvantaged populations, are at risk of making cancer prevention decisions based on incomplete or uninformed preferences [40,41]. This can potentially lead to poor decision quality, lower uptake of cancer prevention strategies, and poorer health outcomes. Digital PtDAs for cancer prevention have the potential to support socially disadvantaged women by providing information that is tailored, understandable, accessible anytime and anywhere, thereby facilitating their engagement in SDM with healthcare professionals. The findings of this scoping review will have important implications for clinical practice, patient empowerment, and future research aimed at optimizing digital PtDA development and evaluation to improve cancer prevention in socially disadvantaged women.

Strengths of our review will identify existing knowledge gaps and opportunities to improve digital PtDAs and better address the needs, preferences and circumstances of socially disadvantaged women in the context of cancer prevention. By informing the development and evaluation of more inclusive and responsive interventions, the review may contribute to improve equity in social determinants of health described by the World Health Organization (WHO) [42]. By including multiple cancer types, this review will provide a comprehensive overview of the current evidence on digital PtDAs for cancer prevention targeting socially disadvantaged women. This broad perspective may be valuable for researchers seeking to better understand the state of the field and identify priorities for future investigation. The findings may also inform the work of healthcare professionals, academics, policymakers, and PtDA developers involved in the design, implementation, and evaluation of interventions that support informed decision-making in cancer prevention.

Limitations to this review include multiple criteria and definitions of a social disadvantage selected within the PROGRESS-Plus framework [34] generating heterogeneity among inclusion criteria of studies to select. Further, determining whether a PtDA is specifically designed for, or adequately addresses the needs of, socially disadvantaged women may be challenging, as these needs are diverse and may not always be explicitly reported in the literature.

The intentional focus on primary and secondary prevention for all cancer types means a broad inclusion of study and likely heterogeneity of the studies included since they may focus on different cancer types and content. Moreover, focusing exclusively on digital PtDAs could potentially hinder the representativeness of world regions characterized by limited digital access and health literacy.

### Dissemination and amendments

The scoping review findings will be disseminated at national and international conferences and published in an open-access international peer-reviewed journal. We will also disseminate findings through patient/citizen and advocacy organizations, professional organizations and healthcare delivery systems, social media, and lay press. We will record any amendments to this protocol in the Open Science Framework (OSF) and report them in the final manuscript.

## Conclusion

By mapping the existing evidence and identifying critical knowledge gaps, this scoping review will provide a foundation for the design, adaptation, and implementation of digital PtDAs that are responsive to the needs of socially disadvantaged women. The implications of the review findings will be useful for clinical practice, patient empowerment, and future research priorities related to the development and integration of digital PtDAs in cancer prevention. It may support more equitable access to informed decision-making in cancer prevention and contribute to reducing health inequities associated with social determinants of health, as described by the WHO [42].

## Supporting information

S1 File Preferred Reporting Items for Systematic reviews and Meta-Analyses extension for Scoping Reviews (PRISMA-ScR) Checklist. (PDF)

S2 File Search strategy. (PDF)

S3 File Data extraction content. (PDF)
