# Supplementary material for: Digital patient decision aids for cancer prevention in socially disadvantaged women: A scoping review protocol
Source: PLoS One. 2026 Oct 14;21(10):e0359332. doi: 10.1371/journal.pone.0359332 (PMC13667833; doi:10.1371/journal.pone.0359332)
Supplement: Supplementary file 2 [file pone.0359332.s002.pdf]

Supporting information 1

Search strategy

A comprehensive search strategy was developed and executed for Embase.com; Medline Ovid SP (via PubMed interface); CINAHL (EBSCO); Cochrane Library Wiley; CENTRAL - Cochrane Library Wiley; Web of Science (Core collection), with adapted syntax in the search field of the database. To allow for transparency, peer review, and reproducibility, the full search strategy, including all keywords and MeSH terms, Boolean operators, truncation, planned limits (e.g., language and publication date), and the number of records retrieved, is presented below:

(exp Decision Making/ OR Physician-Patient Relations/ OR exp Patient Participation/ OR exp Health Communication/ OR exp Patient-Centered Care/ OR exp Decision Support Techniques/ OR Decision Support Systems, Clinical/ OR Patient Education as Topic/ OR Educational Technology/ OR Health Education/ OR Patient Education Handout/ OR (physician-patient relation* OR doctor-patient relation* OR patient decision-making OR share* decision-mak* OR making decision* OR informed decision* OR decision aid* OR decision support method* OR decision-making technique* OR (decision adj3 (aids OR strat* OR method* OR technique* OR intervention* OR tool*)) OR (decision-making ADJ3 (program* or approach*))).mp.)

AND (Healthcare Disparities/ OR Health Status Disparities/ OR Social Determinants of Health/ OR Vulnerable Populations/ OR exp Socioeconomic Factors/ OR Working Poor/ OR Poverty/ OR Minority Health/ OR exp Minority Groups/ OR Race Factors/ OR exp "Sexual and Gender Minorities"/ OR Educational Status/ OR exp Information Literacy/ OR exp Computer Literacy/ OR Literacy/ OR Health Literacy/ OR Language Tests/ OR Medically Underserved Area/ OR

Medically Uninsured/ OR Medical Indigency/ OR Educational Measurement/mt,st OR Multilingualism/ OR Communication Barriers/ OR (((health or healthcare) adj (inequalit* OR disparit*)) OR vulnerab* OR disadvantaged OR "socioeconomic* OR "socio-economic* OR minority group* OR ethnic group* OR "education* level*" OR "level of education" OR education* status OR education* attainment OR health literacy OR low* literacy OR reading abilit* OR reading skill* OR (Rapid estimate adj2 adult literacy) OR wide range achievement test OR underserved OR underinsured OR uninsured OR numeracy OR minority language* OR language barrier*).mp.)

AND (((exp Neoplasms/ OR (neoplas* OR cancer* OR carcinom* OR lymphoma* OR malignan* OR melanom* OR oncolog* OR tumor* OR tumour*).ti,ab,kf.) AND (Preventive Health Services/ OR exp Primary Prevention/ OR Secondary Prevention/ OR Mass Screening/ OR "Early Detection of Cancer"/ OR (prevent* OR prophyla* OR screen*).mp.)) OR Cancer Vaccines/ OR exp Papillomavirus Vaccines/ OR exp Neoplasms/pc OR Papillomavirus Infections/pc OR ((cancer OR HPV OR papillomavirus) ADJ3 vaccin*).mp.)

AND (Algorithms/ OR exp Artificial Intelligence/ OR exp Internet/ OR Internet-Based Intervention/ OR exp Medical Informatics/ OR Information Systems/ OR exp Software/ OR Mobile Applications/ OR ("AI" OR algorithm* OR app OR apps OR "artificial intelligence" OR chatbot* OR "clinical decision support*" OR "computational intelligence*" OR computer* OR digital* OR digitis* OR digitiz* OR ehealth OR e-health OR electronic* OR informatics OR "information system*" OR interactive OR internet OR "machine intelligence*" OR "machine learning" OR mobile OR online OR platform* OR "portable electronic app*" OR "smartphone app*" OR software* OR web).mp.)

The search was conducted in January 2026 and yielded 3,550 references.
