# Supplementary material for: Digital patient decision aids for cancer prevention in socially disadvantaged women: A scoping review protocol
Source: PLoS One. 2026 Oct 14;21(10):e0359332. doi: 10.1371/journal.pone.0359332 (PMC13667833; doi:10.1371/journal.pone.0359332)
Supplement: Supplementary file 4 [file pone.0359332.s005.docx]

**Point-by-point response to academic Editor and Reviewer**

Dear Dr Essouma,

Dear Mr Reynolds,

Thank you for the opportunity to revise our manuscript. We have found the comments helpful, well explained with fair consideration and have strengthened the manuscript. We strove to address them completely and hope that our changes are satisfactory.

Sincerely,

Emmanuelle Lüthi

**1. Academic Editor comments: Please ensure that your manuscript meets PLOS ONE's style requirements, including those for file naming.**

**Author response**: Thank you for your advice and the links, we have reviewed the PLOS ONE’s style requirements including the file names.

**2. Thank you for stating the following in the** **Competing Interests section**: *[Marie-Anne Durand has contributed to the development of Option Grid patient decision aids. EBSCO Information Services sells subscription access to Option Grid patient decision aids. She receives consulting income from EBSCO Health. No other competing interests declared. There is no conflict of interest for the other authors.].*

**Please confirm that this does not alter your adherence to all PLOS ONE policies on sharing data and materials, by including the following statement: "****This does not alter our adherence to PLOS ONE policies on sharing data and materials.” (as detailed online in our guide for authors <http://journals.plos.org/plosone/s/competing-interests>).**

**If there are restrictions on sharing of data and/or materials, please state these. Please note that we cannot proceed with consideration of your article until this information has been declared.**

**Please include your updated Competing Interests statement in your cover letter; we will change the online submission form on your behalf.**

**Author response**: Thank you for this great point, we confirm that we fully agree with PLOS ONE policies on sharing data and materials. We have included the suggested statement in the Competing Interests section, (lines 284-285, p*.*14*)*:

*“Marie-Anne Durand has contributed to the development of Option Grid patient decision aids. EBSCO Information Services sells subscription access to Option Grid patient decision aids. She receives consulting income from EBSCO Health. No other competing interests declared. This does not alter our adherence to PLOS ONE policies on sharing data and materials.*

*There is no conflict of interest for the other authors.”*

We have also updated our Competing Interests statement in the cover letter.

**3. Please provide a complete** **Data Availability Statement in the submission form,** **ensuring you include all necessary access information or a reason for why you are unable to make your data freely accessible. If your research concerns only data provided within your submission, please write "All data are in the manuscript and/or supporting information files" as your Data Availability Statement.**

**Author response**: Thank you for this comment. We have added a Data availability statement in the submission form. As proposed by our research support team, we have created a data repository. All data are available on the Unisanté data repository at the following address: https://doi.org/10.16909/DATASET/69 ». We will also upload raw data in the future.

**4. Please include captions for your Supporting Information files at the end of your manuscript, and update any in-text citations to match accordingly. Please see our Supporting Information guidelines for more information: http://journals.plos.org/plosone/s/supporting-information.**

**Author response**: Thank you for pointing this out. We have added captions for our Supporting Information Files at the end of our manuscript (lines 394-395, p.16):

*“Supporting information*

*S1 File. Search strategy.*

*S2 File. Data extraction content.”*

**Additional Editor Comments:**

**1. Conform to PLOS ONE guidelines for formatting manuscripts. For instance, the maximum word count should be 300 in the abstract, and the abstract should not be subdivided into sub-sections. Furthermore, sections and sub-sections of the manuscript should not be numbered.**

**Author response**: Thank you for pointing this out. We have modified the format of the manuscript accordingly.

**2. Consider thorough language editing to improve the readability of the manuscript.**

**Author response**: Thank you for your advice. The manuscript has been reviewed by a senior medical doctor and researcher, who is an English native speaker and by a bilingual researcher.

**3. Consider delineating the dissemination plan and the OSF reference for the registered protocol in the abstract.**

**Author response**: Thank you for this advice. We have added the dissemination plan and the OSF reference in the abstract as follows (lines 41-45, p.2-3):

“*Findings will be submitted for publication in an international peer-reviewed journal and presented at national and international conferences focused on cancer prevention, shared decision-making, digital health or socially disadvantaged women. We will share findings with healthcare professionals, public health and cancer prevention stakeholders, and socially disadvantaged communities and their network. This protocol has been registered with the Open Science Framework (https://osf.io/f3428/).*”

**4. The longest section of the manuscript should be the "Materials and Methods" section. The current introduction is very long and this extended length may distract readers from the manuscript's goal. Because the topic "cancer" is relatively well known in health research and there is mounting evidence on PtDAs (as shown by the multiple articles cited in your cover letter), I suggest streamlining the introduction section by providing answers to three questions (in max 1 to 1.5 pages): i)** **what is the current knowledge about digital PtDAs use for cancer prevention in women (including socioeconomically disadvantaged women)? ii)** **what are gaps in knowledge aboutthe use of digital PtDAs in women (including socioeconomically disadvantaged women)? iii)** **why do you wish to conduct a scoping review to fill the gap in knowledge (when there are various other forms of data synthesis methods such as systematic reviews) and** **what are the research questions, main review goal, and specific review objectives? The currently proposed research question, main review goal and specific review objectives are unclear to me. This may be due to a language barrier. For the research question, did you wish to write something like: "What are the characteristics of digital PtDAs used in socioeconomically disadvantaged women for cancer prevention, their frequency of use in those women for cancer prevention, and the impact of their use for cancer prevention in those women? A main review goal matching with that question could be: map the body of evidence on digital PtDAs used in women socioeconomically deprived for cancer prevention, their frequency of use for cancer prevention in those women for cancer prevention, and the impact of their use for cancer prevention in those women. Three specific objectives would correspond to that goal: i) determine the characteristics of digital PtDAs used for cancer prevention in socioeconomically disadvantaged women, ii) determine the frequency of use of digital PtDAs for cancer prevention in women socioeconomically disadvantaged, iii) determine the impact of the use of digital PtDAs for cancer prevention in women socioeconomically disadvantaged. You would also need to provide details on the type of impact/effect of digital PtDAs use you will be looking for: impact on cancer prevention at the individual and population levels? Impact on the overall health (physical, social, mental) of individuals of the target population (socioeconomically disadvantaged women) besides the impact on cancer prevention?**

**The articles mentioned in the cover letter should be cited in the introduction section.**

**Author response**: Thank you for this feedback and the pertinent review, we fully agree. We have rewritten and shortened the introduction section to improve clarity. We have reorganized the structure according to the three questions suggested above. We hope our changes will be satisfactory.

We have also simplified the research question, main review goal and specific review objectives. However, we prefer to use the term “socially disadvantaged women” for consistency with the terminology used in the literature reviewed and cited, instead of “socioeconomically disadvantaged women”. Further, our review does not focus on PtDA frequency of use but rather on characteristics, design and impact. Impact of a PtDA could be, for example, improving shared decision making, screening uptake and knowledge about cancer prevention. The impact will depend on the outcomes and results of each included study, detailed in our data extraction table.

We have now incorporated most of the articles referenced in the cover letter into the Introduction, where relevant to the logical flow and key topics addressed in this section.

**5. In agreement with the preceding comment, the research questions and objectives should be moved from the "Materials and Methods" section to the end of the introduction section.**

**Author response**: Thank you for this advice. We have moved the research questions and objectives to the end of the introduction section as proposed (lines 98-109, p.6)

**6. The "Materials and Methods" section is currently difficult to read. Instead of making a long text just for inclusion and exclusion criteria, I suggest combining both the inclusion and exclusion criteria in a sub-section termed "Eligibility criteria", and summarising the eligibility criteria in a Table as did these authors:https: //doi.org/10.1371/journal.pone.0322753. The text in lines 195-199 should be mentioned at hhe beginning of the "Materials and Methods" section, just before the "Eligibility criteria" sub-section, and the time schedule for the completion of the review should be amended taking into account the fact that we are already in July 2026. The text in lines 242-244 belongs to the sub-section "Eligibility criteria". Consider uploading a template of the proposed data extraction sheet as a supplemental material to this submission so that readers can have a clear idea of which data are going to be extracted. The text in lines 266 to 268 belongs to the Discussion section. Consider adding a "****Patient and public involvement" sub-section at the end of the "Materials and Methods" section.**

**Author response**: Thank you for your advice and pertinent review. We have created a Table as suggested. We now refer to it as Table 1. Further, we have moved the text in lines 195-199 (lines 111-117, p.6), in lines 242-244 (lines 122-124, p.7) and in lines 266-268 (lines 212-215, p.10-11) as suggested. We have added a Patient and public involvement section (lines 189-198, p.10):

“*This scoping review is the first phase in a broader four-steps project aimed at developing a digital PtDA for cancer prevention for socially disadvantaged women. To ensure meaningful engagement of the target population throughout the research process, two citizen partners with lived experience of migration are members of the project steering committee. They contribute to project governance through monthly meetings, during which they co-review study materials, participate in key decisions regarding project identity and communication (e.g., project name and logo), co-develop participant recruitment strategies, and co-facilitate meetings with study participants. Their active and sustained involvement helps ensure that the project remains relevant, acceptable, and responsive to the needs and preferences of the intended users.*”

The timeline for the completion of the review has been updated (lines 114-115, p.6):

” *Following the development of the search strategy (September 2025 to January 2026), study and source-of-evidence selection will be conducted between February and August 2026.”*

We have created a supporting information file (S2 file) with the data extraction sheet as suggested.

**7. The Discussion section should be entirely revised. While revising the discussion, reiterate the review goal and objectives in the first paragraph, then state the expected strengths and limitations (eg,** **limited representativeness of the world regions where digital access and literacy is severely limited) of the planned scoping review in the subsequent paragraph, then comes the "Disseminations and amendment" statement.**

**Author response**: Thank you for your comment. We have reiterated the review goal and objectives in the first paragraph of the Discussion and reorganized the subsequent paragraphs as suggested. It now reads (lines 200-206, p.10):

*“We conducted this scoping review to map and synthetize the body of evidence on digital PtDAs for cancer prevention (characteristics, design and impact) targeting socially disadvantaged women. Our review question focused on three main objectives: a) To identify and describe the characteristics of existing digital PtDAs for socially disadvantaged women in cancer prevention, using the IPDAS minimal criteria[28] and the TIDieR checklist [29]; b) To examine the development process, including stakeholder involvement and use of frameworks or guidelines; c) To summarize the evaluation methods and the type of impact reported for these PtDAs.”*

**8. Consider adding a Conclusion section after the "Dissemination and amendments" section of the Discussion section.**

**Author response**: Thank you for this comment. We have added a Conclusion after the "Dissemination and amendments" section of the Discussion. It now reads (lines 243-249, p.12): “*By mapping the existing evidence and identifying critical knowledge gaps, this scoping review will provide a foundation for the design, adaptation, and implementation of digital PtDAs that are responsive to the needs of socially disadvantaged women. The implications of the review findings will be useful for clinical practice, patient empowerment, and future research priorities related to the development and integration of digital PtDAs in cancer prevention. It may support more equitable access to informed decision-making in cancer prevention and contribute to reducing health inequities associated with social determinants of health, as described by the WHO.”*

**Reviewers' comments:**

**Have the authors described where all data underlying the findings will be made available when the study is complete?**

**The PLOS Data policy requires authors to make all data underlying the findings described in their manuscript fully available without restriction, with rare exception, at the time of publication. The data should be provided as part of the manuscript or its supporting information, or deposited to a public repository. For example, in addition to summary statistics, the data points behind means, medians and variance measures should be available. If there are restrictions on publicly sharing data—e.g. participant privacy or use of data from a third party—those must be specified.**

**Reviewer #1: No**

**Author response**: as mentioned in Editor comment’s number 3 (p.3), our research support team recommended that we create a data repository. All data are now available on the Unisanté data repository at the following address: https://doi.org/10.16909/DATASET/69». We will also use this repository in the future to upload future raw data of the scoping review results.

**In the abstract, at lines 39 and 45 you state that the review will be conducted according to the JBI guidelines and reported according to PRISMA, as you should (and is all too commonly incorrect even in published reviews) but in the Data Analysis and Presentation section at line 264 you state that “The review will be designed, conducted, and reported in accordance with the PRISMA extension for scoping reviews (PRISMA-ScR) guidelines” - which should only say it will be reported according to PRISMA-ScR and designed and conducted according to JBI. You do mention following JBI at line 195, at the beginning of the methods. Consider moving the reporting guideline to the beginning of the methods also since it applies to the whole project, not just the data acquisition.**

**Author response**: Thank you for the positive feedback, and the pertinent review. We have made the suggested changes (lines 111-117, p.6):

*“The proposed scoping review will be designed and conducted in accordance with the JBI methodology for scoping reviews [31] and reported according to the PRISMA extension for scoping reviews (PRISMA-ScR) guidelines [32]. Following the development of the search strategy (September 2025 to January 2026), study and source-of-evidence selection will be conducted between February and August 2026. Data extraction, analysis and synthesis are expected to be completed within the subsequent 12 months. This review protocol has been registered in the Open Science Framework (OSF) registry (registration number: f3428) [33].”*

**In the introduction at line 58, you mention tobacco, obesity, and inactivity rates without a citation, where you cite rates for mortality, prevention strategies and effectiveness, and screening rates. Consider citing a source for that.**

**Author response**: Thank you for this great point, we fully agree. We have added the following three references in the introduction paragraph (line 53, p.4). They have been published less than ten years ago and well cited.

- **Casetta, B, Videla, A J, Bardach, A, et al.** Association Between Cigarette Smoking Prevalence and Income Level: A Systematic Review and Meta-Analysis. Nicotine Tob Res. 2017;19(12):1401–7. doi:10.1093/ntr/ntw266.
- **Mohammed, S H, Habtewold, T D, Birhanu, M M, et al.** Neighbourhood socioeconomic status and overweight/obesity: a systematic review and meta-analysis of epidemiological studies. BMJ Open. 2019;9(11):e028238. doi:10.1136/bmjopen-2018-028238.
- **O’Donoghue, G, Kennedy, A, Puggina, A, et al.** Socio-economic determinants of physical activity across the life course: A "DEterminants of DIet and Physical ACtivity" (DEDIPAC) umbrella literature review. PLOS ONE. 2018;13(1):e0190737. doi:10.1371/journal.pone.0190737.

**In the inclusion criteria, at line 131, and elsewhere you refer to screening rates in Switzerland, and elsewhere you focus on Europe. This seems a little inconsistent. Either is fine, but it may be better to choose one. All of Europe or the EU may be more generalizable, but there may be reasons to use the national level, also. If you do need to use both, briefly explain why.**

**Author response**: Thank you for this comment. We felt that starting with Switzerland, where the study was conducted, provided important context before expanding the discussion to a broader European perspective that may be of greater interest to an international readership. However, in line with the reviewer’s suggestion, we have substantially reduced the length of the Introduction. As a result, the concern raised has been addressed and is no longer applicable to the revised manuscript.

**At line 66, is the word “post” missing – pre-encounter and post-encounter?**

**Author response:** Thank you for this comment. Indeed, the word “post” was missing. As the Introduction section has been shortened, these words have been removed.

**In the instruction, the discussion about chatbots would benefit by describing the kind of bot you will include – “smart” AI or LLM powered ones, those with that follow a strict script, all of the above? Will you examine patient privacy protections? In the EU, with stricter digital privacy laws, this may be a given, but non-European readers may have concerns. Similarly, chatbots from places with less rigorous privacy laws may be less attractive to some users.**

**Author response:** Thank you for this comment. We will include all of them. We believe that this information describes inclusion criteria. We added this point in Table 1. The examination of patient privacy protection is beyond the scope of this review. Nevertheless, this consideration remains important, and we intend to discuss it in the Discussion section of the manuscript reporting the results of the scoping review.

**At lines 92 and 94 you refer to Owen’s tool and Selmouni’s chatbot. Using a numbered citation style makes these references confusing. Consider making the names more explicitly connected to the tools. It is unclear which chatbot is referred to in line 97.**

**Author response:** Thank you for pointing this out. We have clarified which chatbot is being referred to. The text now reads (lines 67-75, p.4-5): “*Based on a rapid review of the literature that did not specifically focus on women,, we identified a chatbot PtDA to improve participation in cervical cancer screening among socially disadvantaged women in France [19] and a conversational agent to promote informed decision-making regarding prostate cancer screening among African-American men [20]. The evaluation of Owen’s tool showed significant improvements in prostate cancer knowledge, self-efficacy in informed decision-making, and technology usage. The evaluation of Selmouni’s chatbot PtDA for cervical cancer screening suggested that the chatbot not only enhanced knowledge about cervical cancer screening but also significantly boosted motivation to engage in screening practices. Users—including those with limited digital literacy—found the Selmouni’s chatbot innovative, user-friendly, and informative [21].”*

**The description of the aims of the review at the end of the introduction, lines 104-112 are very good, as are the objectives in the beginning of the methods section.**

**Author response:** Thank you for the positive feedback.

**At line 119, it would be helpful to spell out the acronym IPDAS, which is only mentioned previously in the abstract, and there it does not use the acronym.**

**Author response:** Thank you for this comment. We have now defined the IPDAS acronym in the main text. It now reads (lines 105-106, p.6): *“[…] using the International Patient Decision Aid Standards (IPDAS) minimal criteria [29] […]”*

**At line 126, will you include reports on PtDAs or PtDAs themselves, or both? If so, the word reports or a synonym is missing.**

**Author response:** Thank you for pointing this out. We will include both. We have revised the newly created Table 1 to clearly distinguish between PtDAs reports and standalone PtDAs, as follows: “*Reports on PtDAs and PtDAs themselves explicitly designed to meet the needs of socially disadvantaged women according to the PROGRESS-Plus framework, […]”*

**At lines 119-120, you state that you will exclude women without hereditary risks of cancer. Do you mean you will exclude PtDAs or studies on PtDAs that focus on that population? If so why? It seems that the disadvantaged population may be less aware of hereditary cancer risks than more advantaged women and could benefit more from the tools you are researching.**

**Author response:** Thank you for this comment. Yes, we will exclude PtDAs or studies on PtDAs that focus on women **with** hereditary risks of cancer. This population is not included in organized population-based cancer screening programs and therefore falls outside of the scope of our review. Women with hereditary cancer risk require specific screening and risk management approaches that differ from those used in the general population.

**The types of sources at line 155 forward are nicely described, very inclusive, and appropriate, especially the inclusion of PtDAs that have not been described in published research.**

**Author response:** Thank you for the positive feedback.

**In the Context section, I wondered if interviews or questionnaires with providers, or other paper or verbal tools that were then recorded digitally would be included.**

**Author response:** Thank you for this comment. Interviews or questionnaires mentioned in the above comment do not meet the IPDAS definition of a PtDA used in our scoping review. Therefore, they will be excluded. This information is included in the newly created Table 1, under excluded outcomes as follows:

“• *Tools that do not fulfill the minimal IPDAS definition of a PtDA will be excluded, such as interventions that provide general education only, without linking it to a specific healthcare decision; or are designed primarily to promote treatment adherence or to obtain informed consent for a recommended option rather than to support a deliberative choice among alternatives.”*

**For your search strategy, you mention that a librarian/information specialist assisted with the development of the searches. The extent was unclear. If he wrote the searches or was very actively involved, this is a significant participation in the project in the area of data acquisition and he should be listed as a co-author. If he advised , then the acknowledgement is appropriate.**

**Author response:** Thank you for pointing this out. Indeed, we developed the search strategy collaboratively, building on the work of Pittet et al. (2026). For Pittet et al.’s review, our librarian contributed substantially to the development of the search strategy but preferred to appear in the Acknowledgment section and declined to be listed as a co-author. Given that his role and contributions were similar in the present review, we assumed that he preferred to be acknowledged rather than included as a co-author. The reviewer’s comment prompted us to revisit this point and discuss authorship with him again. Following this discussion, he agreed to be listed as a co-author. We have therefore added his name to the author list and removed it from the Acknowledgments section. (lines 250-254, p.12)

**Will the studies found in Pittet et al (2024) be automatically included? Or is it expected that they will all be found by the search strategy and included through the screening process. It is unclear from line 223.**

**Author response:** Thank you for this comment. Studies included in Pittet et al.’s rapid review will be considered for inclusion in our proposed review if they are identified through our search strategy or related searches. Should any relevant studies not be retrieved, which we consider unlikely, we will manually screen the references from Pittet et al.’s review and include any studies that meet our eligibility criteria.

**At line 230, there is a reference to “potentially relevant sources.” Does this mean the titles and abstracts that were given two yes/include votes at the screening phase. I assume so, but you don’t want a reader making assumptions about your methods.**

**Author response:** Thank you for this comment. We have clarified as follows (lines 154-156, p.8): *“Relevant sources selected and discussed by the two independent reviewers will be retrieved in full and their citation details imported into Rayyan.”*

**In your limitations, you mention that including all cancer types may cause a large amount of heterogeneity. The wide range of cancer types could also be seen as a strength of your study, but this is well noted here, nonetheless.**

**Author response:** Thank you for this comment. We agree. We have added this point in the discussion section as a strength (lines 220-224, p.11): *“By including multiple cancer types, this review will provide a comprehensive overview of the current evidence on digital PtDAs for cancer prevention targeting socially disadvantaged women. This broad perspective may be valuable for researchers seeking to better understand the state of the field and identify priorities for future investigation.”*

**The inclusion of the citizen partners is also excellent. It seems to be done rarely but for topics like yours could provide context that clinicians might not have.**

**Author response:** Thank you for the positive feedback.

**Your example search strategy seems comprehensive. Since this is a protocol I did not evaluate it term by term. I did try to copy it and run it, but the pdf formatting and line numbers introduced too many artifacts for it to run successfully.** **It does seem to be written for Ovid Medline while your protocol says you will search PubMed. Either is fine, of course. Please specify in the appendix and match it to the text in the protocol.**

**Author response:** Thank you for the positive feedback and for pointing this out. We have loaded search strategies for each database reviewed on the Unisanté data repository at the following address: https://doi.org/10.16909/DATASET/69 ». We will also upload raw data in the future.
