# Supplementary material for: Digital patient decision aids for cancer prevention in socially disadvantaged women: A scoping review protocol
Source: PLoS One. 2026 Oct 14;21(10):e0359332. doi: 10.1371/journal.pone.0359332 (PMC13667833; doi:10.1371/journal.pone.0359332)
Supplement: Supplementary file 5 [file pone.0359332.s006.docx]

**Point-by-point response to academic Editor and Reviewer**

Dear Dr Essouma,

Dear Mr Reynolds,

Thank you for the opportunity to improve the minor revision to our manuscript. We have found these new comments helpful. We strove to address them completely and hope that our changes are satisfactory.

Sincerely,

Emmanuelle Lüthi

**Academic Editor comments:**

**1. Please review your reference list to ensure that it is complete and correct. If you have cited papers that have been retracted, please include the rationale for doing so in the manuscript text, or remove these references and replace them with relevant current references. Any changes to the reference list should be mentioned in the rebuttal letter that accompanies your revised manuscript. If you need to cite a retracted article, indicate the article’s retracted status in the References list and also include a citation and full reference for the retraction notice.**

**Author response**: Thank you for this comment. According to your journal guidelines, we have modified the number of authors listed (from 3 to 6) and the order of some of the information for the references of book chapters. We have added the DOI for reference 17 (Elwyn et al. 2006).

**2. In line 205, you wrote that the search strategy was developed between September 2025 and January 2026. Therefore, the text in lines 261-278 should be written using the past tense. Similarly, if the backward citation search has been completed as well, the text in lines 279-283 should also be written using the past tense.**

**Author response**: Thank you for this comment. We have changed the tense of these two paragraphs, as follows (lines 128-149, p.9-10): “*A three-step search strategy aimed to locate both published and unpublished studies. First, an initial limited search of MEDLINE and CINAHL (EBSCO) was undertaken to identify articles on the topic. The words contained in the titles and abstracts of relevant articles, and the Medical Subject Headings (MeSH) and index terms used to describe the articles were used to develop a full search strategy in MEDLINE (S1 File). The search strategy was developed by the research team with the help of an information specialist. The search strategy, including all identified MeSH terms and keywords, was adapted for each database and/or information source. The databases to be searched will included MEDLINE* ***ALL (Ovid)****, CINAHL Complete (****EBSCO****), Embase.com, Web of Science (Core collection), Cochrane Database of Systematic Reviews (****Wiley****) and Cochrane Central Register of Controlled Trials (****Wiley****). To ensure a comprehensive coverage, gray literature and online resources such as the online inventory of Decision Aids developed by the Ottawa Hospital Research Institute [38] and the MAGIC app (https://www.magicevidence.org/magicapp/) were included. The first five pages of Google search results were screened, along with relevant websites of health organizations, government agencies, and patient advocacy groups. Additionally, the first 200 results from a targeted Google Scholar search were reviewed. The search strategy for Google Scholar was developed and refined in collaboration with the information specialist.*

*To identify any additional relevant studies not captured in the database searches, we conducted a backward citation search of the reference lists of all included full-text articles and relevant reviews. This backward citation searching helped capture additional studies that meet inclusion criteria and relevant gray literature or earlier foundational works. Any newly identified citations were screened according to the same eligibility criteria used in previous steps.”*

**Reviewers' comments:**

**Thank you for the opportunity to review this revised scoping review protocol.**

**This protocol seems to be in excellent shape and has only three very minor points that need correction or clarification.**

1. **At lines 67-75 on page 4-5 the mention of the names Owen and Selmani with no prior reference, other than a numbered citation, still seems unclear to me, even with the additional context about the tools they developed. This is probably a matter of style and editing is up to you**

**Author response**: Thank you for pointing this out. We have clarified by adding the names of the first author following the description of the tools at the beginning of the paragraph. It now reads (lines 67-76, p.4-5): “*Based on a rapid review of the literature that did not specifically focus on women, we identified a digital chatbot-based PtDA to improve participation in cervical cancer screening among socially disadvantaged women in France,* ***developed by Selmouni et al.*** *[19] and a conversational agent to promote informed decision-making regarding prostate cancer screening among African-American men,* ***developed by Owen et al.*** *[20]. The evaluation of Owen’s tool showed significant improvements in prostate cancer knowledge, self-efficacy in informed decision-making, and technology usage. The evaluation of Selmouni’s chatbot PtDA for cervical cancer screening suggested that the chatbot not only enhanced knowledge about cervical cancer screening but also significantly boosted motivation to engage in screening practices. Users—including those with limited digital literacy—found Selmouni’s chatbot innovative, user-friendly, and informative [21].”*

2. **At line 131 and 134 the text says you will search Medline via PubMed, while the actual search strategy used Ovid. Please reconcile.**

**Author response:** Thank you for this comment. We fully agree. To correct and avoid any confusion, we have adapted as follows (lines 129; 132 and 136, p.9) : “MEDLINE ALL (Ovid)”.

3. **At line 136, Cochrane CENTRAL is available on both Wiley and Ovid platforms. The data repository indicates you used Wiley. Add this to the text of the protocol to comply with PRISMA-S.**

**Author response:** Thank you for pointing this out. We agree with your comment. We have now added more details. It now reads: “*The databases to be searched will included MEDLINE* ***ALL (Ovid)****, CINAHL Complete (****EBSCO****), Embase.com, Web of Science (Core collection), Cochrane Database of Systematic Reviews (****Wiley****) and Cochrane Central Register of Controlled Trials (****Wiley****).*”

**Overall, this is an excellent protocol. Good luck with the scoping review.**

**Author response:** Thank you for your positive feedback and for your support throughout the revision process, which strengthened the manuscript.
